# Chemotactic and Angiogenic Potential of Mineralized Collagen Scaffolds Functionalized with Naturally Occurring Bioactive Factor Mixtures to Stimulate Bone Regeneration

**DOI:** 10.3390/ijms22115836

**Published:** 2021-05-29

**Authors:** Henriette Bretschneider, Mandy Quade, Anja Lode, Michael Gelinsky, Stefan Rammelt, Corina Vater

**Affiliations:** 1 University Center of Orthopaedic, Trauma and Plastic Surgery, University Hospital Carl Gustav Carus of Technische Universität Dresden, Fetscherstraße 74, 01307 Dresden, Germany; henriette.bretschneider@uniklinikum-dresden.de (H.B.); stefan.rammelt@uniklinikum-dresden.de (S.R.); 2 Centre for Translational Bone, Joint and Soft Tissue Research, University Hospital Carl Gustav Carus and Faculty of Medicine of Technische Universität Dresden, Fetscherstraße 74, 01307 Dresden, Germany; mandy.quade@tu-dresden.de (M.Q.); anja.lode@tu-dresden.de (A.L.); michael.gelinsky@tu-dresden.de (M.G.)

**Keywords:** growth factor, bone regeneration, collagen scaffold, bone substitute materials, MSC secretome, hypoxia-conditioned medium, platelet lysates, adipose tissue extract

## Abstract

To develop cost-effective and efficient bone substitutes for improved regeneration of bone defects, heparin-modified mineralized collagen scaffolds were functionalized with concentrated, naturally occurring bioactive factor mixtures derived from adipose tissue, platelet-rich plasma and conditioned medium from a hypoxia-treated human bone marrow-derived mesenchymal stem cell line. Besides the analysis of the release kinetics of functionalized scaffolds, the bioactivity of the released bioactive factors was tested with regard to chemotaxis and angiogenic tube formation. Additionally, functionalized scaffolds were seeded with human bone marrow-derived mesenchymal stromal cells (hBM-MSC) and their osteogenic and angiogenic potential was investigated. The release of bioactive factors from the scaffolds was highest within the first 3 days. Bioactivity of the released factors could be confirmed for all bioactive factor mixtures by successful chemoattraction of hBM-MSC in a transwell assay as well as by the formation of prevascular structures in a 2D co-culture system of hBM-MSC and human umbilical vein endothelial cells. The cells seeded directly onto the functionalized scaffolds were able to express osteogenic markers and form tubular networks. In conclusion, heparin-modified mineralized collagen scaffolds could be successfully functionalized with naturally occurring bioactive factor mixtures promoting cell migration and vascularization.

## 1. Introduction

Critical size bone defects caused by trauma, implant-related complications, tumor resection or infection represent an unsolved problem in orthopedic and trauma surgery. Due to their intrinsic osteogenic potential and the absence of the risk of immunological rejection, bone autografts are the gold standard for the repair of such defects. However, the limited amount of autologous bone that can be harvested and the inherent donor-site morbidity strongly restrict this therapeutic approach [1]. The tissue engineering concept provides an alternative treatment option: A porous scaffold consisting of a biodegradable bone substitute is colonized with autologous (stem) cells. Their osteogenic differentiation and maturation are stimulated by osteoinductive factors. However, the classical concept of seeding and maturation of these constructs in vitro is limited by the lack of vascularization that results in fast cell death in the inner scaffold regions after implantation [2]. Thus, a promising approach is to functionalize a porous bone replacement material with signaling factors, which are able to attract and stimulate cells from the surrounding host tissue after implantation, promoting the ingrowth of osteogenic cells into but also the formation of a vascular network within the implant [3,4,5]. The key challenges of this in situ tissue engineering approach are to identify suitable signaling factors and to ensure their efficient integration into a suitable bone replacement material as well as their sustained release.

Because bone regeneration comprises multiple biological processes, single factors are less efficient than combinations of different factors exploiting synergistic effects [6,7,8]. However, even in combination, the application of recombinant growth factors requires unphysiologically high concentrations that are cost-intensive and may cause unwanted side effects [9,10,11]. Therefore, various natural sources of bioactive factors have been investigated [12,13,14,15].

Among them, platelet-rich plasma (PRP) has been used for many years to promote wound healing and tissue regeneration in orthopedic, maxillofacial and plastic surgery. With respect to bone repair, the published results are inconsistent: while some studies show a positive effect on bone healing, others show no significant benefit [16]. Thus, the efficacy of PRP and other platelet concentrates in promoting bone regeneration is at the center of a recent academic debate [17,18].

In our recent work, we compared PRP-derived human platelet lysate (PL), conditioned medium from hypoxia-treated human mesenchymal stem cells (HCM) and human adipose tissue extract (ATE) as potential bioactive factor mixtures. We observed significant stimulatory effects of PL and HCM on the chemoattraction of human bone marrow-derived mesenchymal stromal cells (hBM-MSC) as well as of HCM and ATE on osteogenic differentiation of hBM-MSC and angiogenesis [19]. In order to utilize the stimulatory properties of these different bioactive factor mixtures for a possible clinical application, the aim of the present work was to integrate them into scaffolds consisting of a suitable bone replacement material and enable efficient loading with and sustained release of the factors.

Mineralized collagen type I is an artificial bone matrix produced in a biomimetic process of synchronous collagen fibril reassembly and mineralization with nanocrystalline hydroxyapatite [20]. It can be processed into 3D scaffolds with an interconnecting porosity, which allows the ingrowth of cells and prevascular structures [21,22,23]. The suitability of this material to support bone regeneration has been demonstrated in vitro in cell studies with hBM-MSC [22,24] and in vivo in bone defect models of rats and sheep [25,26]. In order to enhance its protein binding capacity, heparin can be efficiently integrated into mineralized collagen either during its synthesis (‘in situ’) or after the scaffold fabrication process (‘post’) [27,28]. The highly sulfated, highly anionic glycosaminoglycan heparin is known to bind various growth factors specifically, stabilizing them within a biomaterial and retarding their release [29,30,31,32]. For mineralized collagen scaffolds modified with heparin, an enhanced binding capacity has been demonstrated exemplarily for the vascular endothelial growth factor (VEGF); moreover, the release rate of VEGF could be controlled by the amount of heparin integrated into the scaffold [28].

In the study presented here, heparin-modified scaffolds of mineralized collagen were functionalized with concentrated bioactive factor mixtures from (i) PL, (ii) HCM and (iii) ATE; (iiii) a mixture (Mix) of PL, HCM and ATE in equal parts was investigated as an additional group in order to test if a further enhancement can be achieved by the combination. The regenerative potential of the functionalized scaffolds was evaluated with respect to chemotaxis, osteogenesis and angiogenesis.

## 2. Results

**Figure 1:**
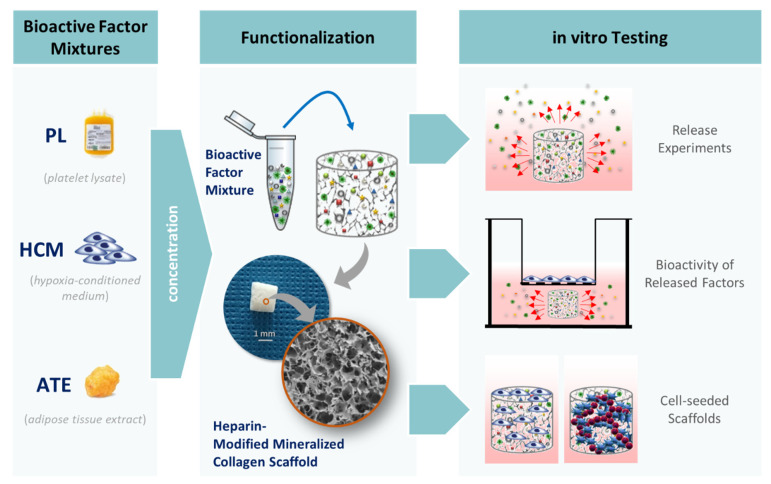
A schematic diagram of the different experiments. Bioactive factor mixtures were produced from platelet lysates (PL), conditioned medium from hypoxia-treated human telomerase immortalized bone marrow-derived mesenchymal stem cells (HCM) and adipose tissue extracts (ATE). Following functionalization of heparin-modified mineralized collagen scaffolds, release kinetics, the bioactivity of the released factors and the response of directly seeded cells were investigated.

Figure 1 provides an overview of the experimental work conducted in this study. Bioactive factor mixtures were derived (i) from platelet-rich plasma through repeated freeze–thaw cycles (PL), (ii) by collecting conditioned medium from a hypoxia-treated human bone marrow-derived mesenchymal stem cell line (HCM) and (iii) by extraction from adipose tissue (ATE). In order to achieve the highest possible protein concentration in the scaffolds, which is also reliably detectable by enzyme-linked immunosorbent assay (ELISA) after release from the scaffolds, the bioactive factor mixtures were concentrated before loading the scaffolds. Heparin-modified mineralized collagen scaffolds were used to achieve a delayed release of proteins and cytokines that extends beyond the very initial phase of bone regeneration. The results of the different experiments for characterization of the scaffold system, including determination of release kinetics, evaluation of bioactivity of released factors and analyses with cell-seeded scaffolds, are presented in detail below.

### 2.1. Release of Bioactive Factors from Functionalized Mineralized Collagen Scaffolds

**Figure 2:**
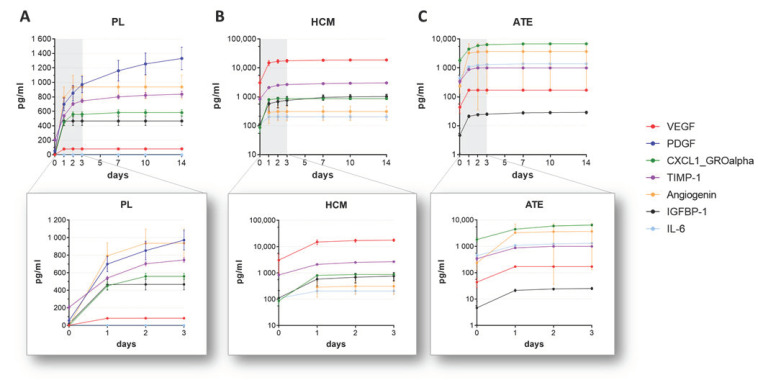
Growth factor and cytokine release from (**A**) PL-, (**B**) HCM- and (**C**) ATE-functionalized mineralized collagen scaffolds over 14 days (upper row) and detailed over 3 days (lower row). Different scaling of the Y-axis was used to ensure suitable data presentation for each factor mixture (mean ± SD, *n* = 3).

The release kinetics of bioactive factors from mineralized collagen scaffolds were analyzed by immersion of the functionalized scaffolds in cell culture medium over 14 days and quantification of various factors in the supernatants taken at defined time points by ELISA. The data revealed a burst release for all 3 tested bioactive factor mixtures with the highest bioactive factor concentrations within the first 3 days (Figure 2). Comparing the 3 mixtures regarding the overall release of bioactive factors, it was highest for PL- followed by ATE- and HCM-functionalized scaffolds. For single factors, such as platelet-derived growth factor (PDGF) and tissue inhibitor of metalloproteinases 1 (TIMP-1), a delayed release was observed over a period of 14 days (Figure 2A, Table S1).

### 2.2. Biological Activity of Released Factors

#### 2.2.1. Chemotaxis

The chemoattractive potential of the bioactive factors released from the functionalized mineralized collagen scaffolds was tested by a membrane-based migration assay (transwell assay). After incubating the functionalized scaffolds for 3 d with 1 mL of release medium, 200 µL of the supernatants containing the released factors were added to the lower chamber of the transwell, and the number of hBM-MSC migrating from the upper towards the lower chamber was determined.

**Figure 3:**
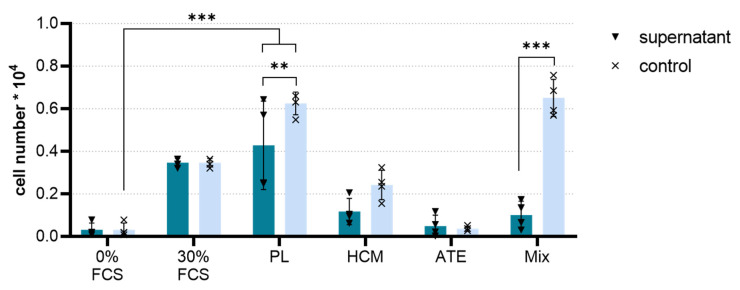
Transwell migration assay. The number of hBM-MSC migrated within 24 h towards supernatants containing released factors from the functionalized scaffolds (dark blue bars) or towards control solutions containing the same amount of bioactive factors that were added to the scaffolds (representing a 100% release; light blue bars) as determined by lactate dehydrogenase (LDH) activity measurement (mean ± SD, *n* = 4 as represented by black triangles and crosses within bars, 2-way ANOVA following Tukey’s post hoc tests: ** *p* < 0.01, *** *p* < 0.001; 0% FCS: negative migration control, 30% FCS: positive migration control).

It was shown that for all bioactive factor mixtures, the chemoattractive potential was maintained after the release of the factors from the scaffolds (Figure 3). In this experimental setup, the PL-functionalized scaffolds showed a significantly higher chemoattractive potential compared to the negative control (medium with 0% FCS, *p* < 0.001), which was also slightly higher than the positive control (medium with 30% FCS). Functionalization with ATE, HCM and the Mix resulted in a significantly lower chemoattractive potential than the positive control, whereby ATE showed the lowest potential in terms of chemoattractivity in our experiment. Except for ATE, cell migration towards the control solutions was increased as compared to the respective scaffold supernatants.

#### 2.2.2. Angiogenic Potential

**Figure 4:**
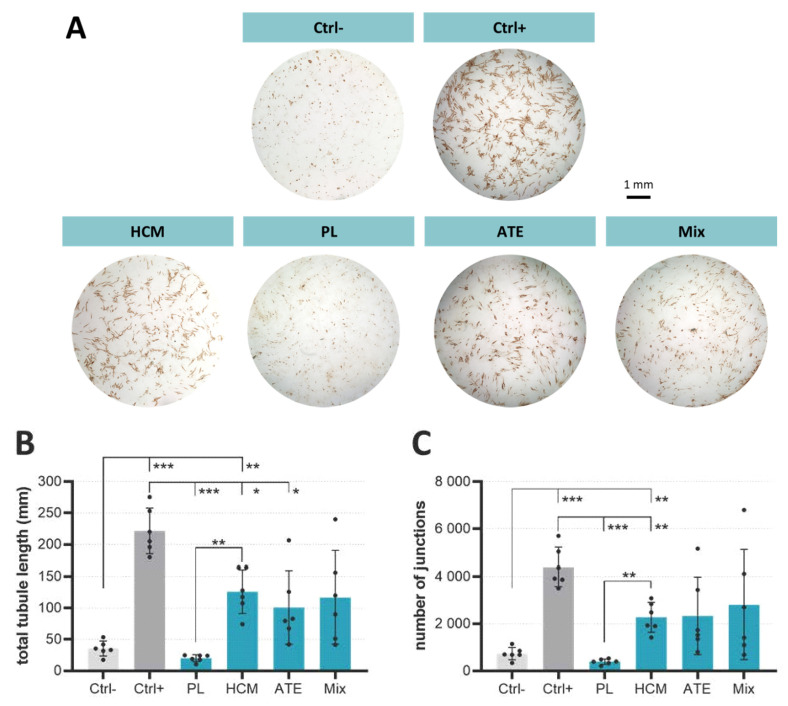
Angiogenic potential of supernatants containing released factors from the functionalized scaffolds as analyzed by a co-culture of hBM-MSC and HUVEC. (**A**) Light microscopic images of prevascular structures visualized by CD31 immunostaining after 7 days of cultivation, (**B**) total tubule length and (**C**) number of junctions (mean ± SD, *n* = 6 as represented by black dots within bars, Welch’s ANOVA following Dunnett’s T3 post hoc tests: * *p* < 0.05, ** *p* < 0.01, *** *p* < 0.001; Ctrl−: co-culture medium without bioactive factor mixtures/VEGF, Ctrl+: co-culture medium with 20 ng/mL VEGF).

The angiogenic potential of supernatants taken from the functionalized scaffolds after 2 days and containing released factors of PL, HCM, ATE or Mix was compared by co-cultivation of hBM-MSC and human umbilical vein endothelial cells (HUVEC) for 7 days. As proven by CD31 staining, supernatants of scaffolds functionalized with HCM, ATE and Mix were able to induce the formation of prevascular structures (Figure 4A). Compared to the negative control (Ctrl−), detailed analyses of these structures revealed longer tubules and an increased number of junctions when cells were treated with supernatants from HCM-, ATE- and Mix-functionalized scaffolds; the increase was significant for HCM (Figure 4B,C). Supernatants derived from PL-functionalized scaffolds neither had an effect on total tubule length nor on the number of junctions when compared to the negative controls.

### 2.3. Analysis of Cell-Seeded Functionalized Scaffolds

#### 2.3.1. Osteogenic Potential—Cell Number Increase, Specific ALP Activity and Gene Expression of Osteogenic Markers

**Figure 5:**
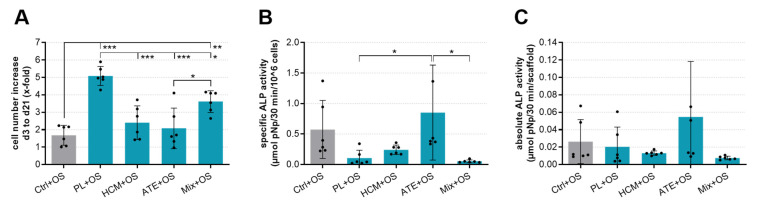
The cell number increase and osteogenic differentiation of hBM-MSC cultured on functionalized mineralized collage scaffolds. Cell-seeded scaffolds without bioactive factor mixture functionalization served as Ctrl (Ctrl+OS). (**A**) Cell number increase from day 3 to day 21, (**B**) specific and (**C**) absolute ALP activity after 21 days of cultivation (mean ± SD, *n* = 6 as represented by black dots within bars, 1-way ANOVA following Tukey’s post hoc tests: * *p* < 0.05, ** *p* < 0.01, *** *p* < 0.001; OS: osteogenic supplements).

hBM-MSC were seeded onto the functionalized mineralized collagen scaffolds and cultivated for up to 21 days in a cell culture medium containing osteogenic supplements (OS). LDH and alkaline phosphatase (ALP) activity were measured. Compared to the control (Ctrl+OS, scaffold without functionalization), PL or Mix functionalization of scaffolds led to a significant cell number increase of hBM-MSC during 21 days of cultivation (Figure 5A). HCM + OS and ATE + OS were significantly inferior to PL + OS in this respect. In contrast, no significant differences between the groups were observed concerning specific ALP activity (Figure 5B).

**Figure 6:**
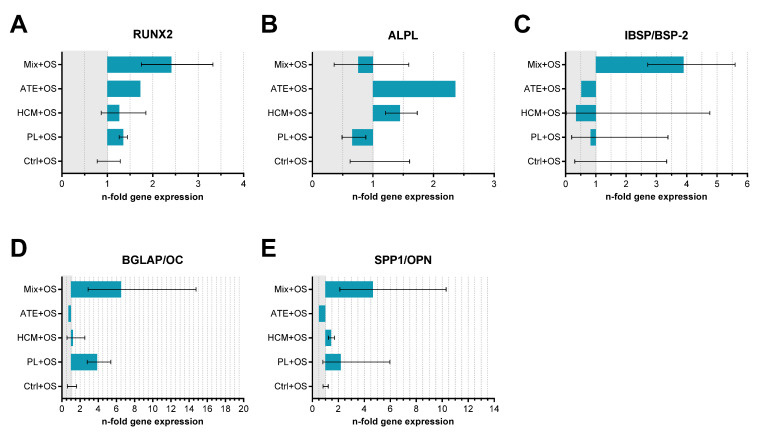
Relative gene expression (*n*-fold of Ctrl+OS) of osteogenic markers (**A**) RUNX2, (**B**) ALPL, (**C**) IBSP/BSP-2, (**D**) BGLAP/OC and (**E**) SPP1/OPN in hBM-MSC cultured with osteogenic supplements in cell culture medium for 21 days on functionalized mineralized collagen scaffolds. Cell-seeded scaffolds without bioactive factor mixture functionalization served as control (Ctrl+OS; mean ± upper and lower limits, *n* = 2).

Gene expression analysis revealed a trend of increased expression of transcription factor RUNX2 (runt-related transcription factor 2), IBSP (bone sialoprotein), BGLAP (osteocalcin) and SPP1 (osteopontin) in cells cultured on Mix-functionalized scaffolds. Similar to the biochemical measurement, there were no significant differences concerning the ALPL (alkaline phosphatase) expression (Figure 6).

#### 2.3.2. Angiogenic Potential—Endothelial Tube Formation

**Figure 7:**
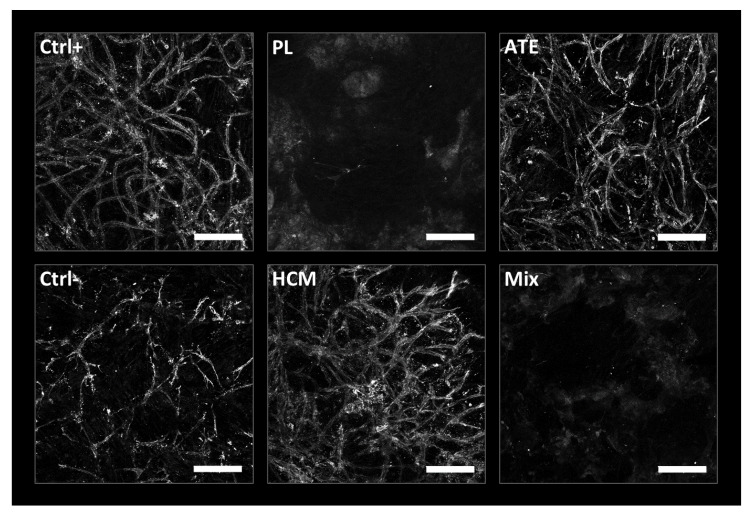
Prevascular structures formed by co-culture of hBM-MSC and HUVEC on functionalized mineralized collagen scaffolds (CD31 staining, laser scanning microscopy, scale bar = 200 µm).

The angiogenic potential of the functionalized scaffolds was evaluated by seeding hBM-MSC in co-culture with HUVEC on top of the scaffolds and culturing them for 10 days. CD31 staining of prevascular structures revealed that HCM and ATE stimulated the formation of an endothelial network, which was qualitatively comparable to the positive control (medium with 20 ng/mL VEGF). In contrast, on scaffolds functionalized with PL and Mix, no clear endothelial tubular structures were observed (Figure 7).

## 3. Discussion

The aim of this study was to investigate a scaffold-based approach for a potential clinical application of bioactive factor mixtures derived from platelet lysate (PL), hypoxia-conditioned medium (HCM) and adipose tissue extract (ATE), which has been previously reported to exert chemoattractive, angiogenic and/or osteogenic effects [19]. Prior to loading onto the scaffolds, the bioactive factor mixtures were concentrated to ensure sufficiently high and effective concentrations of the factors after their release. Scaffolds containing heparin were used to enhance the binding capacity as well as a sustained delivery of the factors [27,28].

As shown in the previous study by protein and cytokine array analysis [15], the bioactive factor mixtures contain a wide variety of growth factors, chemo- and cytokines. For analysis of the release experiments, proteins and cytokines that are present in high amounts and important in the context of chemotaxis and angiogenesis were selected for ELISA. Because of the different biological sources and production processes (Table 1), there were substantial differences in the concentrations of the bioactive factors in the respective mixture and their release. Despite heparin modification of the scaffolds, a burst release within the first 3 days of incubation could be observed for nearly all analyzed bioactive factors independently of the mixture group. In the case of VEGF, after 3 days, 57% of the loaded amount was already released from PL-functionalized scaffolds, followed by 60% from HCM- and 71% from ATE-functionalized ones. Since there was no considerable VEGF release from day 3 until day 14, it seems to be likely that the remaining amount of VEGF was retained by the scaffold and would be released only when the scaffold is resorbed (e.g., by osteoclasts in vivo) or degrades. Similar results were found for VEGF-functionalized calcium phosphate bone cements [33,34]. The burst release observed in our study is in contrast to investigations performed by Knaack et al., where modification of the mineralized collagen scaffolds with heparin led to a more sustained release of VEGF over 8 days [28]. Here, using the same amount and process of heparin modification (“post,” 30 mg/g collagen), after 3 days, only about 9% of the initially loaded VEGF were released. One explanation is that in the present study, a mixture of factors was loaded onto the scaffold, whereas Knaack et al. used (recombinant) VEGF only. Within a mixture, it can be assumed that the different factors compete for free binding sites at the scaffold and that the binding efficiency of a single factor is reduced by the presence of the high protein content within the bioactive factor mixture. Thus, the binding and release of the factors are not only dependent on their chemical interaction with the scaffold but also with each other. The only growth factor showing a sustained release from the scaffolds is PDGF, which is present in detectable amounts only in PL. After 14 days of incubation, only about 38% of the initially loaded PDGF were released, indicating a continued release.

The bioactivity of the released factors was investigated with regard to chemotaxis in a transwell migration assay and angiogenesis in an hBM-MSC/HUVEC co-culture model. Compared to the negative control (0% FCS), the migration of hBM-MSC was significantly stimulated only by supernatants of PL-functionalized scaffolds (*p* < 0.001); supernatants of the HCM and Mix group showed a low, non-significant chemotactic effect, whereas supernatants of the ATE group possessed no chemotactic potential (Figure 3). These observations are largely in line with the results obtained for the bioactive factor mixtures in the scaffold-free approach recently described [19], albeit the bioactivity of the supernatants of HCM- but also of PL-functionalized scaffolds seems to be somewhat reduced. The direct comparison of the chemotactic effect of the supernatants to those of control solutions, which contain the same amount of bioactive factors loaded onto the scaffolds, confirmed the reduced bioactivity of the supernatants. This reduction was significant in the case of the PL and Mix groups but not in the case of the HCM group (Figure 3). The most plausible explanation is that after 3 days of incubation, at least some of the factors were only partially released from the scaffolds (Figure 2; Table S1), and therefore, the supernatants contained a lower factor concentration. For example, the release of PDGF, which is known for its chemotactic effect and appears prominent in PL (Table S1), continued beyond 3 days [35,36]. Moreover, the ratio between various factors in the mixture might have changed after release by the different release kinetics, and this could affect synergistic effects. A partial loss of bioactivity as a result of the concentration procedure or binding to the mineralized collagen scaffolds is unlikely. For HCM, we could demonstrate that dialysis, freeze-drying and reconstitution of the mixture did not reduce the bioactivity. Furthermore, complete maintenance of bioactivity after binding to and release from mineralized collagen scaffolds has been demonstrated for VEGF applied as a single recombinant factor [23,28].

An unexpected outcome was the low chemotactic potential of the supernatants derived from the Mix-functionalized scaffolds. The combination of several bioactive factors was assumed to provoke an increase in chemoattraction in comparison to the single mixture. However, chemoattraction by Mix supernatants seemed to be inhibited instead (Figure 3). Based on the fact that such a reduction in the chemotactic potential was not observed for the control solution (containing the same amount of bioactive factor mixture loaded onto the scaffolds), a competition of the different factors of PL, ATE and HCM for binding sites at the scaffolds and finally a reduced loading efficiency can be assumed. An inhibitory effect of the combination per se does not seem to be plausible because, for the control solution of Mix (containing only 1/3 of PL), a comparably high number of migrated cells has been observed as for the control solution of PL. This indicates that the additional presence of HCM (and maybe even ATE) could have been contributed to the chemotactic potential.

In conclusion, the transwell migration assay indicated maintenance of the chemotactic potential of bioactive factor mixtures obtained from PL and HCM. For ATE-derived mixtures, no chemotactic effects were observed, which is in line with our previous findings [19]. For ATE, a stimulatory effect on angiogenesis and stem cell differentiation has been reported before [19,37]. At least in the set up investigated in the present study, the combination of the bioactive factor mixtures in the Mix group is not beneficial with respect to enhanced cell attraction.

In order to study angiogenic effects in an hBM-MSC/HUVEC co-culture model, cells were either incubated with supernatants from functionalized scaffolds or directly seeded onto them. Compared with the negative control, supernatants from PL-functionalized scaffolds showed no stimulatory effect on the formation of prevascular structures. In general, this can be explained by the low abundance of angiogenic factors in PL [15]; e.g., after 2 days of incubation, only 0.082 ng/mL VEGF was released (Table S1). Accordingly, when cells were seeded directly onto PL-functionalized scaffolds, no prevascular structures were visible. Since PL also contains fibrinogen, as a result of physiological coagulation, fibrin is formed in contact with calcium-containing media [38]. This effect becomes prominent if PL is added systemically and could be seen in a previous study [19]. Here, analysis of angiogenesis was not possible due to fibrin accumulation in the well plate covering the cells. In the present study, no fibrin accumulation could be observed in the 2D assay using PL supernatants, indicating that the fibrinogen present in PL is strongly bound or adsorbed onto the scaffolds, converted into fibrin and not released within 2 days.

In the case of HCM, both the supernatants and functionalized scaffolds themselves stimulated angiogenesis. In the 2D assay, total tubule length and the number of junctions (Figure 4) were significantly increased when compared to the negative control. Nevertheless, despite comparable VEGF concentrations (17 ng/mL in supernatants after 2 days of incubation vs. 20 ng/mL VEGF in the positive control), the total tubule length and the number of junctions were significantly lower than the positive control indicating a reduced bioactivity of the released factors. Another explanation is that the angiogenic effect is not mainly mediated by VEGF but by the synergistic interaction of the different factors present in HCM, which might not have been released in a sufficient amount within the 2 days. This is confirmed by a study by Quade et al. where the systemic application of diluted HCM containing only 2.5 ng/mL VEGF led to significantly longer tubules and higher numbers of junctions [23]. In direct contact with HCM-functionalized scaffolds, cells formed prevascular structures that were comparable to that of the positive control (Figure 7). This could already be observed in former studies in response to recombinant VEGF [27] and HCM released from a central depot within scaffolds of mineralized collagen [23].

Angiogenesis was also stimulated by ATE–both in the presence of supernatants from and directly on the functionalized scaffolds. This is in line with a report by Sarkanen et al., who incorporated ATE into a hyaluronan hydrogel and demonstrated a gradual release of the bioactive factors as well as microvessel formation and adipose tissue deposition when implanted subcutaneously into rats [37]. Although total tubule length and number of junctions were comparable between HCM and ATE supernatants, for ATE, they were not significantly different from the negative control due to high standard deviations (Figure 4). In this context, it is worth mentioning that the VEGF concentration within ATE supernatants after 2 days of incubation was only 1/100 of that found in HCM supernatants (Table S1; HCM: 17 ng/mL vs. ATE: 0.17 ng/mL) but leading to a comparable formation of prevascular structures. This can also be seen in the 3D assay where hBM-MSC and HUVEC were directly seeded onto the scaffold (Figure 7) and strengthens the hypothesis that stimulation of angiogenesis is less a question of concentration but rather a question of the optimal composition of the bioactive factors.

In terms of total tubule length and number of junctions, supernatants from Mix-functionalized scaffolds were comparable to that from HCM- and ATE-functionalized ones (Figure 4). Since Mix-functionalized scaffolds contain only 1/6 and 1/12 of the amount of bioactive factors loaded onto HCM- and ATE-functionalized ones (Mix: 1/3 of 10× HCM/ATE vs. HCM: loaded with 20× HCM and ATE: loaded with 40× ATE), a synergistic effect of the included factors is probable. Like for PL, no formation of prevascular structures was visible when cells were seeded directly onto Mix-functionalized scaffolds. This might be due to the adsorption of fibrinogen to the scaffold surface following conversion to fibrin and therefore preventing microscopic analysis as seen for PL samples. Nevertheless, keeping in mind that prevascular structures had been formed when cells were exposed to supernatants from Mix-functionalized scaffolds, it is likely that they are formed also on the scaffolds but could not be detected by the staining and microscopic methods used. With regard to angiogenesis, it can be concluded that factors released from HCM-, ATE- and Mix-functionalized scaffolds are able to stimulate angiogenesis, which can also be seen when cells are in direct contact with the scaffolds.

The impact of the scaffold functionalization with the 4 bioactive factor mixture groups on cell number increase and osteogenic differentiation of hBM-MSC, seeded onto the scaffolds, was studied in the presence of osteogenic supplements (+OS) since after implantation into a bone defect in vivo, the implants are exposed to osteostimulatory factors, too. In this way, also inhibitory effects of the bioactive factor mixtures should be discovered. However, a limitation of these experiments was that the osteogenic supplements used in the in vitro set up were artificial. In terms of cell number increase, HCM and ATE functionalization did not influence the cell behavior in comparison to the control; however, a significantly higher cell number increase during the 21 days cultivation period was observed on scaffolds functionalized with PL. The intermediate value determined for the Mix group, albeit still significantly higher than the control, indicated an “averaging” of the 3 combined bioactive factor mixtures and did not hint at either a synergistic or an inhibitory effect of this mixture combination on cell number increase (Figure 5A).

The specific ALP activity (ALP activity related to cell number) was not significantly influenced by the scaffold functionalization with all bioactive factor mixture groups (Figure 5B). However, the high standard deviations caused by the usage of primary hBM-MSC from 2 different donors make a clear interpretation of the data difficult. Thus, a stimulatory effect of ATE and an inhibitory effect of PL and HCM cannot be excluded. The presentation of the absolute ALP activity (ALP activity per scaffold), as well as the ALPL gene expression analysis, support the hint at a stimulatory effect of ATE in this experimental set up. On the other side, gene expression analyses of IBSP, BGLAP and SPP1 indicated rather an inhibitory effect of ATE, albeit the differences to the control were again not significant (Figure 5C and Figure 6). Surprisingly, the expression of RUNX2, IBSP, BGLAP and SPP1 was clearly, but not significantly enhanced in the Mix group compared to the other bioactive factor mixture groups as well as the control (Figure 6). In conclusion, the analyses of hBM-MSC-seeded scaffolds indicated a significant stimulation of cell number increase by PL and Mix functionalization but not by HCM and ATE functionalization, whereas no significant stimulation of osteogenic differentiation has been observed for all bioactive factor mixture groups. This outcome confirmed the results of the scaffold-free approach [19] for PL but not for HCM and ATE, indicating that the 3D environment of mineralized collagen scaffolds might have considerably influenced the effect of the bioactive factor mixtures.

The findings of this study have to be seen in light of some limitations. First, scaffold dimensions were chosen with regard to future in vivo testing in a murine femoral bone defect model [39]. Therefore, the scaffolds used for this study were quite small, and therefore, the loading volume of bioactive factor mixtures was limited to 10 µL, which might have not been sufficient to detect significant differences. The second limitation concerns the kind of cells used for the cell culture experiments. Depending on the experiment, primary hBM-MSC of only 2–3 donors were used, leading to high donor variations as seen by high standard deviations and therefore might not reflect the general population. In future studies, this could be improved by using more cell donors when performing the experiments that might lead to more homogeneous and clearer results. Lastly, bioactive factor mixture-functionalized scaffolds were tested in vitro using artificial systems that can only partly reflect their suitability for bone regeneration. Further studies are warranted to find the optimal composition of bioactive factors needed to stimulate chemotaxis, angiogenesis and osteogenesis.

In summary, with this study, we could prove the chemotactic and angiogenic effect of mineralized collagen scaffolds functionalized with the bioactive factor mixtures. Since osteogenesis is coupled with chemotaxis of (stem) cells and angiogenesis [40,41,42], the functionalized scaffolds might stimulate but are probably not sufficient as sole adjuvants to induce efficient bone regeneration. Thinking about the clinical application of these scaffolds for regeneration of bone defects, one possibility could be to combine the bioactive factor mixtures with an additional osteogenic stimulus or factor such as the bone morphogenetic protein 2 (BMP-2) [43]. Thereby, unphysiological high concentrations of BMP-2 as used today and its negative side effects might be avoided.

Additionally, a future aim would be to evaluate the osteoregenerative potential of optimized bioactive factor mixture-functionalized scaffolds in vivo.

## 4. Conclusions

In the current study, the regenerative potential of heparin-modified mineralized collagen scaffolds functionalized with naturally occurring bioactive factor mixtures was evaluated with respect to chemotaxis, osteogenesis and angiogenesis. Our data demonstrate that functionalization of the scaffolds with PL, HCM, ATE or a mixture of them leads to potential bone replacement materials promoting cell migration and vascularization. However, further in-depth studies are needed to elucidate the osteoregenerative potential of bioactive factor mixture-functionalized implants in an in vivo model to confirm our findings.

## 5. Materials and Methods

### 5.1. Cells

Primary hBM-MSC were isolated from bone marrow aspirates of 4 donors (3 male, age 24–33; 1 female, age 60; all Caucasian), expanded in α-MEM/GlutaMAX (ThermoFisher Scientific, Waltham, MA, USA) containing 15% FCS (Corning, NY, USA) and 100 U/mL penicillin/100 µg/mL streptomycin (Pen/Strep; ThermoFisher Scientific) and used in passage 3 and 4 for the experiments. hBM-MSC was characterized according to the criteria of the International Society for Cellular Therapy (ISCT) [44]. The use of hBM-MSC was approved by the institutional review board (ethics committee) of the Technische Universität Dresden. Human umbilical vein endothelial cells (HUVEC) were purchased from Promocell (Heidelberg, Germany), expanded in Endothelial Cell Growth Medium (Promocell) and used in passage 4 for the experiments.

A human mesenchymal stem cell line expressing human telomerase reverse transcriptase (hTERT-MSC) [45], kindly provided by Matthias Schieker (Laboratory of Experimental Surgery and Regenerative Medicine, University Hospital Munich (LMU), Munich, Germany), was used for the HCM production to obtain reproducible HCM batches without the variations caused by using primary cells of individual donors.

### 5.2. Generation and Concentration of Bioactive Factor Mixtures

Platelet-rich plasma lysate (PL), hypoxia-conditioned medium (HCM) and adipose tissue extract (ATE) were generated as described previously [19]. Briefly, PL was produced by repeated freezing/thawing of human platelet-rich plasma (10 expired platelet concentrates, 25 individual donors in total, provided by the German Red Cross, BSD Ost, Dresden, Germany). HCM was received by culturing hTERT-MSC under hypoxic conditions (1% O_2_, 5% CO_2_, 37 °C) for 5 days in Dulbecco’s modified Eagle’s Medium (DMEM) without phenol red (ThermoFisher Scientific) containing 2% human serum following dialysis against ddH_2_O. To generate ATE, adipose tissue was harvested from 5 healthy human donors undergoing a reconstructive operation after informed consent (approval of the Institutional Review Board, protocol number 263122004) and incubated with DMEM without phenol red (24 h, 37 °C, 5% CO_2_). After filtration (40 µm filter) and centrifugation (2000 rpm, 10 min), the supernatants were dialyzed against ddH2O and sterile filtered (0.22 µm filter). Until used, the bioactive factor mixtures were stored at −80 °C.

PL, HCM and ATE were characterized by the quantification of the total protein content as well as by cytokine and angiogenesis protein arrays and ELISA of selected growth factors and cytokines, as previously reported [19] (Table 1).

To achieve the highest possible protein concentration in the scaffolds, which is also reliably detectable by ELISA after release from the scaffolds, the bioactive factor mixtures were concentrated before loading the scaffolds. Therefore, the mixtures were freeze-dried under sterile conditions and resuspended in saline (0.9% NaCl, Fresenius, Germany) up to the solubility limit. Due to the different protein contents within the mixtures, a 10-fold concentration of PL, a 20-fold concentration of HCM and a 40-fold concentration of ATE were possible. In addition, after freeze-drying and reconstitution, a mixture (Mix) of PL, HCM and ATE in equal parts, each concentrated 10-fold, was prepared and used for scaffold functionalization, too.

### 5.3. Heparin-modified Mineralized Collagen Scaffolds—Preparation and Functionalization

Mineralized collagen scaffolds were produced and modified with heparin (30 mg/g collagen) as described previously [27]. In brief, collagen type I from the bovine tendon (kindly provided by Syntacoll, Hessen, Germany) dissolved in hydrochloric acid was mixed with calcium chloride solution; the pH value was adjusted to 7.0 by the addition of TRIS and phosphate buffer. During incubation at 37 °C for 12 h, collagen fibril reassembly and precipitation of hydroxyapatite nanocrystals occurred simultaneously. Concentrated mineralized collagen suspension was filled into a 384-well plate, frozen at –20 °C and freeze-dried to create porous scaffolds, which were then cross-linked with EDC (2 wt% 1-ethyl-3-(3-dimethyl aminopropyl) carbodiimide in 80 vol% ethanol, 1 h). After washing and freeze-drying, the scaffolds were incubated in MES buffer (2-(N-morpholino) ethanesulfonic acid; Sigma-Aldrich; pH 5.5) containing heparin (Sigma-Aldrich) at room temperature for 24 h [28]. Finally, the scaffolds were washed, cut, freeze-dried and sterilized by γ-irradiation at 25 kGy. For the functionalization of the mineralized collagen scaffolds (length: 2 mm, Ø: 1 mm, loading capacity: 10 µL), 10 µL of concentrated PL, HCM, ATE or Mix (4.2.) were added to the scaffolds, incubated for 24 h in a humid atmosphere at 37 °C and subsequently either freeze- or air-dried under sterile conditions. For the 3D angiogenesis experiment (see Section 5.5.3), larger mineralized collagen scaffolds without heparin modification (length: 3 mm, Ø: 5 mm, loading capacity: 50 µL) were used and functionalized with 50 µL of concentrated PL, HCM, ATE or Mix. Post-processing of the large scaffolds was the same as for the small ones.

### 5.4. Release Kinetics of Functionalized Mineralized Collagen Scaffolds

#### 5.4.1. Quantification of selected released bioactive factors by ELISA

Functionalized scaffolds were transferred into bovine serum albumin(BSA)-coated 48-well plates (1% BSA in phosphate-buffered saline for 30 min) and incubated with 1 mL/scaffold cell culture medium (DMEM + 10% FCS and 1% Pen/Strep, *n* = 3) for 14 days. Native scaffolds without functionalization served as the negative control. Additionally, as a positive control, 10 µL of concentrated PL, HCM and ATE were directly added to 990 µL of cell culture medium, representing a 100% release from the scaffolds (*n* = 1). During the incubation period, supernatants were collected at day 0 (5 min after adding the medium), 1, 2, 3, 7, 10 and 14, followed by adding 1 mL fresh medium. ELISA was performed according to the manufacturer’s instructions for tissue inhibitor of metalloproteinases 1 (TIMP-1), vascular endothelial growth factor (VEGF), platelet-derived growth factor-BB (PDGF-BB; all from PeproTech GmbH, Hamburg, Germany), angiogenin, insulin-like growth factor-binding protein 1 (IGFBP-1; both from Sigma-Aldrich, St. Louis, MO, USA) as well as for the chemokine (C-X-C motif) ligand 1 (CXCL-1/GROα; R&D, Santa Clara, CA, USA). Absorbance measurements were performed according to the manufacturer’s specifications using a microplate reader (Infinite^®^ M200 Pro, Tecan, Switzerland).

#### 5.4.2. Chemotaxis Assay

The chemoattractive potential of the released bioactive factors was tested using a transwell migration assay (Corning^®^ HTS Transwell^®^-96 permeable supports, pore size: 8.0 μm; Sigma Aldrich). The scaffolds functionalized with PL, HCM, ATE or Mix were incubated in BSA-coated 48-well plates for 3 days with 1 mL of release medium (DMEM without phenol red, FCS and Pen/Strep). 200 μL of the collected supernatants were then added to the lower chamber of the transwell serving as a chemoattractant. DMEM without FCS (0% FCS) was used as negative and DMEM with 30% FCS as positive control. Additionally, same as for the release experiments, 10 µL of concentrated PL, HCM, ATE and Mix were directly added to 990 µL of DMEM, representing a 100% release from the scaffolds.

After being starved in an FCS-free medium for 24 h, 2.5 × 10^4^ hBM-MSC were seeded in 75 μL DMEM without phenol red or other supplements into the upper chamber of the transwell and allowed to migrate for 24 h. Then, medium from both chambers was removed, cells were washed 2× with PBS, and non-migrated cells on top of the membrane were removed with a cotton swab. By adding 200 µL of lysis buffer (1% Triton-X-100/PBS; Sigma Aldrich) to the lower chamber followed by incubation for 1 h at room temperature, the number of cells migrated through the membrane was determined by measurement of lactate dehydrogenase (LDH) activity using a CytoTox96^®^ Non-Radioactive Cytotoxicity Assay (Promega, Madison, WI, USA). Experiments were performed in duplicates with hBM-MSC from 2 different individual donors.

#### 5.4.3. In vitro Angiogenesis Assay

For investigating the effect of the released bioactive factors on angiogenesis, scaffolds functionalized with PL, HCM, ATE or Mix were incubated in BSA-coated 48-well plates for 2 days with a 1 mL co-culture medium. Angiogenesis co-culture medium consisted of a 1:1 mixture of α-MEM/GlutaMAX and Endothelial Cell Basal Medium (Promocell) containing 10% heat-inactivated FCS, 1% Pen/Strep, 100 nM dexamethasone (Dex), 50 µM L-ascorbic acid 2-phosphate (AAP) and 5 mM ß-glycerolphosphate (ß-GP; all from Sigma-Aldrich) that was established as hBM-MSC/HUVEC co-culture medium previously [23]. Next, 6.8 × 10^3^ hBM-MSC were seeded into 96-well plates and cultured for 2 days in α-MEM/GlutaMAX containing 15% FCS and 1% Pen/Strep. Then, the medium was removed, 1.7 × 10^3^ HUVEC resuspended in co-culture medium were seeded on top of the hBM-MSC monolayer and allowed to adhere for at least 4 h [23]. As a next step, 100 μL of the supernatants collected from the functionalized scaffolds were added to each well of the co-culture. Co-culture medium supplemented with 20 ng/mL rhVEGF_165_ (Peprotech, Rocky Hill, NJ, USA) served as positive (Ctrl+) and co-culture medium without VEGF as negative control (Ctrl−). After 7 days of co-culture without medium change, the cells were washed with PBS and fixed in 4% neutral buffered formaldehyde (FA; SAV LP GmbH, Flintsbach am Inn, Germany).

To visualize the formed prevascular structures, fixed samples were washed 3x with PBS, incubated for 30 min with 3% goat serum to block unspecific binding sites, washed 3× with PBS again and incubated for 1 h with a monoclonal antibody against CD31 (mouse anti-human, DAKO, Carpinteria, CA, USA; 1:200 in PBS). After 3× washing with PBS, ZytoChem Plus HRP One-Step Polymer anti-Mouse/Rabbit/Rat (Zytomed Systems GmbH, Berlin, Germany; ready-to-use) was added for 30 min. Then, the samples were washed 3× with PBS and incubated with DAB (3,3′-diaminobenzidine; Zytomed Systems GmbH) for approximately 5–10 min. Widefield microscopy was performed using a BZ-9000 microscope (Keyence, Japan), and the images were analyzed using the ImageJ 1.48t (Wayne Rasband, Kensington, MD, USA) and AngioSys 1.0 (TCS Cellworks, Buckingham, UK).

### 5.5. Experiments to Analyze Cell-Seeded Scaffolds functionalized with Bioactive Factor Mixtures

#### 5.5.1. Cell-Seeding of hBM-MSC to Investigate Osteogenic Potential

Primary hBM-MSC were seeded at 5 × 10^4^ cells per scaffold and cultivated for up to 21 days with medium change every 3 days in DMEM/GlutaMAX (ThermoFisher Scientific) containing 10% FCS, 1% Pen/Strep and osteogenic supplements (OS: 100 nM Dex, 50 µM AAP, 10 mM β-GP). Cell-seeded native scaffolds without functionalization served as control. After 3 and 21 days, cell-seeded scaffolds were washed with PBS and frozen at −80 °C until LDH and alkaline phosphatase (ALP) activity measurement. Experiments were performed in triplicates with hBM-MSC from 2 different donors.

#### 5.5.2. Analysis of LDH and ALP Activity

Frozen samples were thawed on ice for 20 min following incubation with 1% Triton-X-100/PBS (Sigma-Aldrich) on ice for 50 min. LDH activity within the lysates was determined using the CytoTox 96^®^ Non-Radioactive Cytotoxicity Assay according to the manufacturer’s instructions by measuring the absorbance at 490 nm (Infinite^®^ M200 Pro, Tecan, Switzerland). Cell number was calculated by correlation to the LDH activity of defined cell numbers after lysis (calibration line). To determine cell number increase, the cell number detected on day 21 was divided by the cell number detected on day 3 after seeding. ALP activity was determined by incubating an aliquot of the same cell lysate, as used for LDH quantification with 1 mg/mL p-nitrophenyl phosphate (Sigma Aldrich) in 0.1 M diethanolamine, 1% Triton X-100, 1 mM MgCl_2_ (pH 9.8). After incubation for 30 min at 37 °C, the enzymatic reaction was stopped by adding 1 M NaOH and absorbance was measured at 405 nm (Infinite^®^ M200 Pro). ALP activity was calculated by correlating the absorbance to a p-nitrophenol calibration line and expressed as absolute ALP activity per scaffold and as specific ALP activity normalized to the respective cell number determined by LDH activity measurement.

#### 5.5.3. RNA Isolation and Gene Expression Analysis

Total RNA was isolated using the peqGOLD MicroSpin Total RNA Kit (PeqLab, Erlangen, Germany) according to the manufacturer’s instructions. After measuring the RNA concentration (NanoDrop ND-1000 spectrophotometer, PeqLab), RNA was stored at −80 °C until analysis.

The synthesis of cDNA was performed using the High Capacity cDNA Reverse Transcription Kit (ThermoFisher Scientific) with 250 ng of RNA as template and with the following cycling conditions: 10 min at 25 °C, 120 min at 37 °C, 5 min at 85 °C and storing at 4 °C. The cDNA was stored at −20 °C until quantitative real-time polymerase chain reaction (qRT-PCR) analysis.

For PCR reactions, the TaqMan Fast Advanced Master Mix and TaqMan Gene Expression Assays (both ThermoFisher Scientific) for eukaryotic translation elongation factor 1 alpha 1 (EEF1A1), runt-related transcription factor-2 (RUNX2), alkaline phosphatase (ALPL), integrin-binding sialoprotein/bone sialoprotein 2 (IBSP/BSP-2), bone gamma-carboxyglutamate protein/osteocalcin (BGLAP/OC) and secreted phosphoprotein 1/osteopontin (SPP1/OPN) were used according to the manufacturer’s instructions. PCR was run with an Applied Biosystems^®^ 7500 fast Real-Time PCR System (Applied Biosystems, part of ThermoFisher Scientific). All PCR data were analyzed using the ΔΔCt method, normalized to EEF1A1 expression, followed by fold-change calculation and computation of lower (2^−(∆∆Ct + SD_∆Ct)^) and upper (2−^(∆∆Ct − SD_∆Ct)^) limits.

#### 5.5.4. Cell-Seeding of hBM-MSC/HUVEC to Investigate Angiogenic Potential

Angiogenesis within the functionalized mineralized collagen scaffolds was investigated using a co-culture of hBM-MSC and HUVEC. Therefore, dry, functionalized scaffolds (length: 3 mm, Ø: 5 mm) were placed into BSA-coated 48-well plates (1% BSA in PBS, 30 min), seeded with 5 × 10^4^ hBM-MSC in 25 µL αMEM/GlutaMAX + 15% FCS and 1% Pen/Strep and allowed to adhere for 30 min in a humidified atmosphere at 37 °C and 5% CO_2_. Then, 500 µL αMEM/GlutaMAX + 15% FCS and 1% Pen/Strep were carefully added to the wells. After incubation of 2 days, 1.25 × 10^4^ HUVEC in 25 µL Endothelial Cell Growth Medium (Promocell) were seeded on top of the hBM-MSC monolayer and allowed to adhere for at least 4 h. Then, 500 µL/well of co-culture medium (4.4.3) was carefully added, and the cells were cultured until day 10 with medium changes at day 4 and 8. For immunohistochemical analysis of prevascular structures, scaffolds were washed with PBS, fixed with 4% neutral buffered FA for 2–4 h, washed 3× with PBS again, incubated for 30 min with 3% goat serum to block unspecific binding sites, washed 3× with PBS again and incubated for 1 h with a monoclonal antibody against CD31 (mouse anti-human, DAKO; 1:200 in PBS). After washing the scaffolds 3× with PBS, CD31-positive structures and cell nuclei were stained via Alexa Fluor^®^ 546-labelled goat-anti mouse antibody (8 µg/mL; Life technologies, Carlsbad, CA, USA) and Hoechst 33342 (2 µg/mL; Invitrogen, Waltham, MA, USA) for 30 min in the dark and imaged using a Zeiss Axiovert with ApoTome (Zeiss, Oberkochen, Germany).

### 5.6. Statistical Analysis

Assuming a normal distribution of all data, differences between multiple groups were tested using 1- or 2-way analysis of variance (ANOVA) with Tukey’s post hoc testing or, in case of largely differing standard deviations, Welch’s ANOVA with Dunnett’s T3 post hoc testing. All data are presented as mean ± standard deviation. All statistical analyses were performed using GraphPad Prism (Prism 8 for Windows, version 8.4.3 (686); GraphPad Software, San Diego, CA, USA). *p* < 0.05 was set as the limit for statistical differences.

## Figures and Tables

**Table 1:** The total protein content and concentrations of selected bioactive factors within native, unconcentrated platelet lysate (PL), hypoxia-conditioned medium (HCM) and adipose tissue extract (ATE) as used for this study and reported previously [19] (nd: not detectable).

	PL	HCM	ATE
total protein content	11.6 mg/mL	0.015 mg/mL	4.05 mg/mL
IGFBP-1	2905 pg/mL	3026 pg/mL	1417 pg/mL
angiogenin	159 pg/mL	1447 pg/mL	890 pg/mL
TIMP-1	498 pg/mL	1992 pg/mL	924 pg/mL
CXCL1	1970 pg/mL	1469 pg/mL	890 pg/mL
PDGF	8233 pg/mL	nd	60 pg/mL
VEGF	605 pg/mL	12,7378 pg/mL	398 pg/mL
IL-6	nd	804 pg/mL	784 pg/mL

## Supplementary Materials

The following are available online at https://www.mdpi.com/article/10.3390/ijms22115836/s1. Table S1. Daily release of bioactive factors (absolute values) from PL-, HCM- and ATE-functionalized mineralized collagen scaffolds.

## Abbreviations

AAP	Ascorbic acid-2-phosphate
ATE	Adipose tissue extract
bFGF	Basic fibroblast growth factor
CD	Cluster of differentiation
Ctrl+	Positive control
Ctrl−	Negative control
Dex	Dexamethasone
EGF	Epidermal growth factor
ELISA	Enzyme-linked immunosorbent assay
FCS	Fetal calf serum
GP	Glycerophosphate
hBM-MSC	Human bone-marrow-derived mesenchymal stromal cells
HCM	Hypoxia-conditioned medium
HMGB1	High-mobility group protein B1
hTERT-MSC	Human telomerase immortalized bone-marrow-derived mesenchymal stem cells
HUVEC	Human umbilical vein endothelial cells
IGF-1	Insulin-like growth factors-1
IGFBP-1	Insulin-like growth factor-binding protein 1
IL	Interleukin
MSC	Mesenchymal stromal cells
OS	Osteogenic supplements
PDGF-BB	Platelet-derived growth factor BB
PL	Platelet lysate
PRP	Platelet-rich plasma
TGF-ß	Transforming growth factor β
TIMP-1	Tissue inhibitor of metalloproteinases-1
TNFα	Tumor necrosis factor α
VEGF	Vascular endothelial growth factor
