# Supplementary material for: Chemotactic and Angiogenic Potential of Mineralized Collagen Scaffolds Functionalized with Naturally Occurring Bioactive Factor Mixtures to Stimulate Bone Regeneration
Source: Int J Mol Sci. 2021 May 29;22(11):5836. doi: 10.3390/ijms22115836 (PMC8199046; doi:10.3390/ijms22115836)
Supplement: Supplementary file 1 [file ijms-22-05836-s001.zip › ijms-1233767-supplementary.pdf]

Supplementary Material

Table S1. Daily release of bioactive factors (absolute values) from PL-, HCM- and ATE-functionalized mineralized collagen scaffolds. Therefore, functionalized scaffolds were incubated with 1 mL release medium for 14 days with collecting supernatants on day 0, 1, 2, 3, 7, 10 and 14. The same amount of bioactive factor mixtures that was used for functionalization of the single scaffolds (10 µL) were added to 990 µL release medium representing a 100 % control (mean ± SD, *n* = 3; nd: not detectable).

Protein/Cytokin	Concentration in pg/mL		
	**PL**	**HCM**	**ATE**
**VEGF**			
100 % control	142.70	28947.10 ± 3268.77	240.48
d0	2.01 ± 1.68	3075.81 ± 2084.48	43.96 ± 17.12
d1	78.53 ± 7.82	11957.44 ± 975.73	126.73 ± 25.84
d2	0.99 ± 0.88	2054.87 ± 640.63	nd
d3	nd	681.83 ± 321.73	nd
d7	nd	934.20 ± 351.07	nd
d10	nd	214.25 ± 70.91	nd
d14	0.18 ± 1.51	106.84 ± 42.11	nd
**PDGF-BB**			
100 % control	3466.45	nd	nd
d0	54.98 ± 29.15	nd	nd
d1	644.03 ± 63.20	nd	nd
d2	153.59 ± 19.91	nd	nd
d3	119.33 ± 10.08	nd	nd
d7	189.43 ± 29.70	nd	nd
d10	95.13 ± 8.58	nd	nd
d14	75.81 ± 7.58	nd	nd
**Angiogenin**			
100 % control	1098.29	321.04	1261.21
d0	0.05 ± 0.09	nd	235.56 ± 104.22
d1	792.09 ± 147.19	288.47 ± 168.33	3083.63 ± 3561.23
d2	143.34 ± 24.62	23.75 ± 12.03	306.06 ± 144.85
d3	1.85 ± 1.87	0.02 ± 0.02	82.69 ± 90.28
d7	nd	nd	27.31 ± 44.69
d10	nd	nd	nd
d14	nd	nd	nd
**TIMP-1**			
100 % control	399.87 ± 28.94	1037.03 ± 0.77	603.60 ± 10.90
d0	206.31 ± 18.25	838.86 ± 28.96	343.28 ± 45.46
d1	333.40 ± 22.93	1272.73 ± 126.46	540.97 ± 59.88
d2	162.77 ± 3.44	409.06 ± 59.81	116.74 ± 40.88

d3	42.70 ± 10.48	166.88 ± 24.31	2.12 ± 2.05
d7	54.84 ± 5.30	185.45 ± 18.41	nd
d10	22.83 ± 11.24	99.70 ± 15.40	nd
d14	14.70 ± 2.56	79.21 ± 16.47	nd
**CXCL1**			
100 % control	767.91 ± 8.78	1042.44 ± 17.75	2736.66 ± 54.62
d0	nd	86.62 ± 31.05	1832.48 ± 325.80
d1	450.40 ± 42.63	720.13 ± 22.95	2674.45 ± 13.49
d2	108.18 ± 12.74	66.22 ± 14.51	1432.68 ± 262.70
d3	nd	nd	501.41 ± 288.07
d7	24.27 ± 15.74	nd	384.31 ± 201.50
d10	nd	nd	51.23 ± 59.58
d14	nd	nd	9.51 ± 16.48
**IGFBP-1**			
100 % control	709.67 ± 501.67	2307.32	276.89
d0	20.15 ± 10.31	111.52 ± 103.65	4.66 ± 3.57
d1	444.12 ± 54.00	470.83 ± 166.08	16.72 ± 3.44
d2	2.97 ± 0.37	105.94 ± 7.88	2.85 ± 1.13
d3	nd	71.07 ± 13.81	1.07 ± 0.29
d7	nd	198.25 ± 69.31	2.77 ± 0.58
d10	nd	42.55 ± 9.82	0.48 ± 0.11
d14	nd	27.78 ± 2.24	0.48 ± 0.27
**IL-6**			
100 % control	nd	1023.08 ± 108.01	1844.45
d0	nd	108.70 ± 43.55	443.46 ± 112.28
d1	nd	95.97 ± 5.92	649.79 ± 58.44
d2	nd	nd	143.53 ± 35.57
d3	nd	nd	77.88 ± 6.13
d7	nd	nd	77.10 ± 3.84
d10	nd	nd	nd
d14	nd	nd	nd
